# Who is ‘the person of unsound mind’? The problem of terminological incompatibility in law and medical sciences in the context of the proper legal protection of people with mental disorders subjected to penal coercive measures

**DOI:** 10.1192/j.eurpsy.2023.403

**Published:** 2023-07-19

**Authors:** M. Burdzik

**Affiliations:** Department of Psychiatry, Center of Psychiatry in Katowice; Institute of Legal Sciences, University of Silesia, Katowice, Poland

## Abstract

**Introduction:**

Penal coercive measures (e.g., detention) seriously interfere with the individual’s fundamental rights (especially the right to liberty). It is necessary to have proper guarantee mechanisms to protect an individual against the arbitrariness of decisions made in this regard. It is especially significant in the case of people with mental disorders (MD). This group of entities may not be able to take intended legal actions to protect their rights and, thus, requires enhanced legal protection. The effectiveness of legal solutions depends on the appropriate terminology. Vague, ambiguous, or archaic terms pose a risk of over-interpretation and create an area for abuse. An example of such solution is art. 5(1)(e) of the European Convention on Human Rights (ECHR) allows deprivation of liberty for “the person of unsound mind.”

**Objectives:**

The study aims to analyze the concept of “person of unsound mind” appearing in the ECHR and to define its semantic scope in relation to mental disorders. This procedure aims to determine whether the status of a person of unsound mind is the same as the status of a person with MD - both in legal and medical contexts.

**Methods:**

The study consists of two stages. The first stage included the narrative review of the literature by searching the PubMed and Google Scholar databases with the keywords “unsound mind” and “person of unsound mind”. The second stage included the analysis of the European Court of Human Rights judgments relating to art. 5(1)(e) of ECHR, collected in the HUDOC database. Forty-four articles and 128 judgments met inclusion criteria and were included for further analysis.

**Results:**

The study shows that the concept of a “person of unsound mind” is primarily indefinite. The term does not correspond to the current standards of medical terminology. It relates to mental disorders but has a narrower scope. The term “unsound mind” refers only to “true mental disorder”, which is of that kind or degree that warrants compulsory confinement. To be considered a “true” mental disorder has to be of a certain severity. This term should be interpreted narrowly, but there are no grounds to limit its scope to psychotic disorders only. However, including some non-psychotic disorders in its scope may be questionable (e.g., antisocial personality disorder).

**Conclusions:**

The structure of art. 5(1)(e) ECHR does not comply with the current medical terminology standards. This inconsistency in terminology and primary indefinite character of the “unsound mind” may implicate a lot of difficulties in precisely defining its meaning and scope of use in individual cases. It is dangerous from the perspective of the personal liberty of people with MD. This term should be replaced with the term “mental disorders,” the meaning of which is well-established in medicine.

**Disclosure of Interest:**

None Declared

